# Photosensitive nanocarriers for specific delivery of cargo into cells

**DOI:** 10.1038/s41598-020-58865-z

**Published:** 2020-02-07

**Authors:** Pedro Mena-Giraldo, Sandra Pérez-Buitrago, Maritza Londoño-Berrío, Isabel C. Ortiz-Trujillo, Lina M. Hoyos-Palacio, Jahir Orozco

**Affiliations:** 1 Max Planck Tandem Group in Nanobioengineering, Universidad de Antioquia, Calle 67 N° 52-20, Complejo Ruta N, Medellín, 050010 Colombia; 2 Departamento de Ingeniería, Pontificia Universidad Católica del Perú, Av. Universitaria 1801, Lima, Peru; 3 Facultad de Medicina, Grupo de Investigación Biología de Sistemas, Universidad Pontificia Bolivariana, Calle 78 B N°, 72A-109 Medellín, Colombia

**Keywords:** Drug delivery, Biophotonics

## Abstract

Nanoencapsulation is a rapidly expanding technology to enclose cargo into inert material at the nanoscale size, which protects cargo from degradation, improves bioavailability and allows for controlled release. Encapsulation of drugs into functional nanocarriers enhances their specificity, targeting ability, efficiency, and effectiveness. Functionality may come from cell targeting biomolecules that direct nanocarriers to a specific cell or tissue. Delivery is usually mediated by diffusion and erosion mechanisms, but in some cases, this is not sufficient to reach the expected therapeutic effects. This work reports on the development of a new photoresponsive polymeric nanocarrier (PNc)-based nanobioconjugate (NBc) for specific photo-delivery of cargo into target cells. We readily synthesized the PNcs by modification of chitosan with ultraviolet (UV)-photosensitive azobenzene molecules, with Nile red and dofetilide as cargo models to prove the encapsulation/release concept. The PNcs were further functionalized with the cardiac targeting transmembrane peptide and efficiently internalized into cardiomyocytes, as a cell line model. Intracellular cargo-release was dramatically accelerated upon a very short UV-light irradiation time. Delivering cargo in a time-space controlled fashion by means of NBcs is a promising strategy to increase the intracellular cargo concentration, to decrease dose and cargo side effects, thereby improving the effectiveness of a therapeutic regime.

## Introduction

Functional nanocarriers for intracellular drug delivery are systems ideally composed of biodegradable and biocompatible materials such as natural polymers, lipids, amphiphilic polymers, among others, assembled with cell-targeting biomolecules (CTBs)^1,2^. Nanocarriers can be designed for transporting a variety of cargo, either encapsulated into, adsorbed at, or dispersed with the nanocarriers^3^. Encapsulation of therapeutic agents into nanocarriers protects them from degradation, improves their solubility and bioavailability, and enhances the efficiency and effectiveness of therapeutic regimens. However, nanocarrier systems have shown some limitations related to storage stability and administration route, because they are susceptible to aggregation and early degradation^4^. Biodistribution may be also unspecific, generating inefficient therapies, side effects, genetic damage or mutations. To solve these issues, apart from carboxylic, amino, poly(ethylene glycol) and poly(phosphoester) moieties that can be placed at the outermost nanocarrier surface to confer a stealth effect, they can be functionalized with specific CTBs for site-specific controlled cargo release^5–7^. Targeting ability and specificity of the resultant NBcs allows them to accumulate in the target cell or tissue at higher concentrations compared to nanoparticles without activity, thus reducing doses, frequency, toxicity and potential adverse effects that most drugs intrinsically have^8,9^.

Whereas the small size of NBcs, which are commonly less than 200 nm for biomedical applications^10,11^, allows crossing of most physiological barriers in the body, cargo can be delivered at the specific place either by its slow diffusion or by the nanocarrier erosion mechanism. However, these naturally occurring cargo release processes may not be sufficient to reach the required doses of a certain treatment. In this context, encapsulating the cargo into PNcs for its triggered and spatial-temporal release into cells or tissues, through an external stimulus such as irradiation, could dramatically contribute to the improvement of drugs therapeutic effect^12^.

PNcs are based on polymers, which have been chemically modified to contain photoactive groups that may experience structural, conformational or stereochemical changes, such as photocleavage and photoisomerization by exposition to near-infrared (NIR) or UV irradiation^13–16^. Nanocarriers with irreversible photocleavable structures suffer from an alteration of their polarity by UV and NIR irradiation, producing irreversible photo-ruptures of some chromophores from the nanostructures^17^. In contrast, the photoactive groups of reversible photoisomerization nanocarriers suffer a reversible isomerization from cis to trans by UV-absorption, where the trans isomer can be reconverted into the cis isomer by visible light^18^. Such rupture or isomerization processes destabilize the nanostructures, allowing the cargo to be released^19^. Unlike the most biocompatible NIR-based nanocarriers, whose photorelease time is in the order of hours and radiation is dispersed through the tissue^11,20^, the UV light-based nanocarriers, with relatively higher toxicity, require only a few minutes (or even seconds) for cargo photo-delivery^21,22^. In this context, the formulation of the NBcs can be fine-tuned based on the application required.

**Figure 1:**
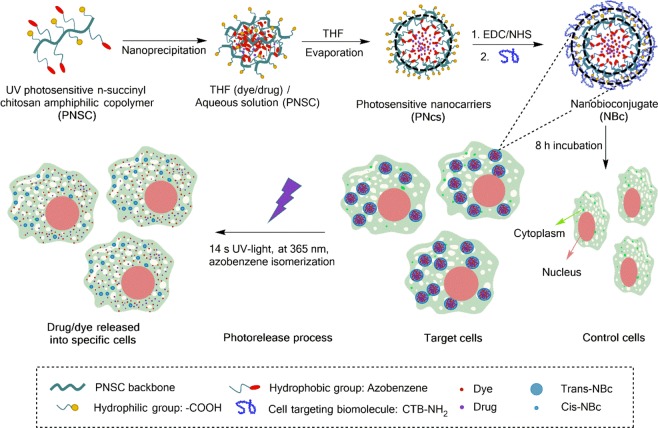
Schematic illustration of PNc synthesis, nanocarrier self-assembly, cargo co-encapsulation and further functionalization with the CTB. Selective internalization of the resultant NBc and UV-light induced cargo release into cardiomyocytes by an isomerization process of the azobenzene photosensitive molecule-containing PNc.

This work reports on the development of a new PNc functionalized with a CTB for rapid and specific photo-delivery of cargo into target cells (Fig. 1). The PNcs were readily synthesized by introducing UV-photosensitive azobenzene molecules into the backbone of the n-succinyl chitosan (NSC) biopolymer while monitoring all the synthesis process steps by Fourier Transform Infrared Spectroscopy (FT-IR) and Nuclear Magnetic Resonance (^1^H NMR). The nanocarriers were further self-assembled and characterized by Transmission Electron Microscopy (TEM), Scanning Electron Microscopy (SEM), Dynamic Light Scattering (DLS) and Electrophoretic Light Scattering (ELS). They were further functionalized with a FITC-labelled CTB and probed to be efficiently internalized into cardiomyocytes, used as a cellular model, as demonstrated by fluorescence microscopy imaging experiments. We used Nile red and dofetilide as drug models to prove the encapsulation/release concept by estimating the intracellular cargo release by UV-vis spectrophotometry. The results show that drug release was dramatically accelerated upon a very short UV-light irradiation time. Cell line viability experiments on both incubation, with the resultant NBc, and UV irradiation demonstrated its harmlessness at the time studied. Controlled and specific delivery of cargo by means of NBcs is a promising strategy to reach therapeutic doses with less cargo concentration, potentially decreasing side effects and improving therapy effectiveness. Indeed, the overall results demonstrated that this proof-of-concept holds considerable promise for the triggered delivery of dofetilide in cardiac cells to fight atrial fibrillation, fulfilling the demand currently requested by the scientific community for this purpose^23,24^.

## Materials and Methods

### Materials

Nile red, 2-Chloroethanol, potassium iodide, N,N-dimethylformamide (DMF), 4-phenylazophenol, potassium carbonate, succinic anhydride, pyridine, acetic ether, hydrochloric acid (HCl), acetic acid (CH_3_COOH), chitosan (medium molecular weight), acetone, sodium hydroxide (NaOH), N,N′-dicyclohexylcarbodiimide (DCC), 1-ethyl-3-(3- dimethylaminopropyl) carbodiimide (EDC), N-hydroxysuccinimide (NHS), tetrahydrofuran (THF), isopropanol, dimethyl sulfoxide (DMSO), 2-[4-(2-hydroxyethyl)piperazin-1-yl]ethanesulfonic acid (HEPES), disodium phosphate (Na_2_HPO_4_), monopotassium phosphate (KH_2_PO_4_), potassium chloride (KCl), tween 20, sodium chloride (NaCl), 3-(4,5-dimethylthiazol-2-yl)-2,5-diphenyltetrazolium bromide (MTT), ethidium bromide, 3,3′-dihexyloxacarbocyanine iodide (DIOC6), DMEM supplemented with 10% (v/v) fetal bovine serum. All reagents were commercially acquired from Sigma-Aldrich (USA). Dofetiled was adquired from Cayman Chemical company. FITC-Cardiac Targeting Peptide (FITC-CTP), sequence: APWHLSSQYSRT, was commercially acquired from the GenScript Company (China). The human fetal liver cells (Hepg2), lung cells (A549) and ventricular cardiomyocytes (RL-14) are commercially available cell lines (American Type Cell Culture Patent Deposit Designation No. PTA-1499, Manassas, VA). The cellular line RL-14 has been established from nonproliferating primary cultures derived from human fetal heart tissues. They used a mitochondrial function-based method to introduce indirectly the SV-40 gene into cells, which are normally intractable to standard transformation techniques^25,26^.

### Photosensitive amphiphilic copolymer synthesis

#### Synthesis of 2-(4-phenylazophenoxy)ethanol (PAPE)

**Figure 2:**
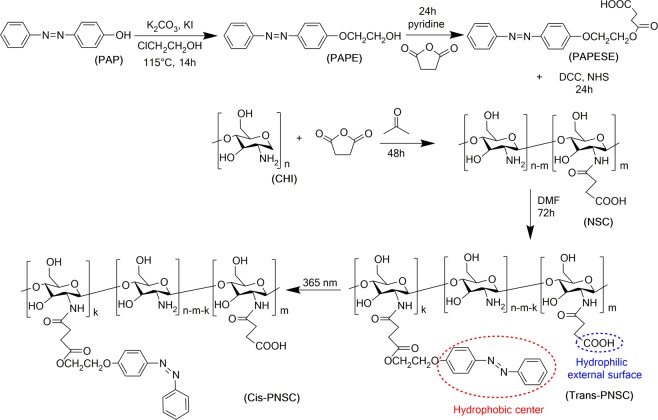
Synthesis and photoisomerization process of PNSC amphiphilic copolymer.

The azobenzene moiety PAPE was obtained by modifying the methodology published by L. Wu *et al*.^27^ A Williamson ether synthesis was carried out in two stages: First, 2-choloroethanol (1.2 ml) and potassium iodide (2.4 g) as catalysts were dissolved in DMF (30 ml) under constant stirring at 70 °C for 20 min. Second, 4-phenylazophenol (PAP) (1.2 g) and potassium carbonate (5.7 g) catalyst were added and the reaction mixture was stirred at 115 °C for 14 h (Fig. 2), under reflux and nitrogen atmosphere. Finally, the catalysts were filtered and the liquid solution was stored to continue the functionalization of the polymer^28^.

#### Synthesis of photoresponsive 2-(4-(phenylazo)phenoxy)ethanoloxy succinyl ester (PAPESE)

PAPE (0.2076 g) and succinic anhydride (0.6204 g) were added to pyridine (10 ml) and reacted in dark by 24 h under constant stirring at room temperature (Fig. 2). Upon completion, the reaction mixture was extracted with acetic ether/HCl removing pyridine from the reaction medium, achieved by several extraction steps. The organic phase was washed with HCl (1 M) for three times to remove pyridine. After that, the mixture was evaporated under vacuum in two stages, first at 300 mbar, 60 °C for 6 h, then the temperature was increased to 130 °C. Finally, the solid PAPESE was removed with a plastic spatula and left in a desiccator for two days.

#### Synthesis of N succinyl chitosan (NSC) and N-succinyl-N-4-(2-(4-(phenylazo)phenoxy)ethanoloxy)-succinyl-chitosan (PNSC)

The natural polymer chitosan (medium molecular weight, 1 g) and succinic anhydride (3 g) were dissolved in acetone (20 ml) and reacted for 48 h at room temperature (Fig. 2). After that, the resultant mixture was precipitated with 4% (w/v) NaOH (40 ml) and then filtered. The precipitate (NSC) was washed with ethanol three times and then dried under vacuum at 60 °C, for 6 h^17^. The activation of the carboxylic acid was carried out by dissolving PAPESE (1 g) in DMF (10 ml) and allowing to react with DCC/NHS (1.5/1.5) during 24 h in dark, under constant stirring. Subsequently, NSC (1 g) solubilized in 5 ml of CH_3_COOH 0.4 M was added to the activated-PAPESE mixture and stirred at room temperature for 72 h (Fig. 2). The copolymer was obtained by precipitation in NaOH 5% (w/v) and collected by filtration, and washed with ethanol three times. Finally, the copolymer PNSC was dried at 60 °C, for 12 h.

#### Characterization and detailed spectral data of the PNSC synthetic route by ^1^H NMR, FT-IR and UV/Vis

**Figure 3:**
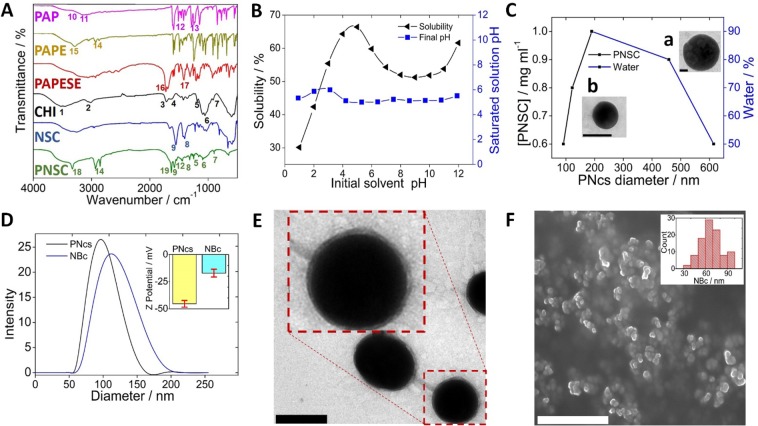
Characterization of the PNSC synthetic route by FT-IR (**A**), the characteristic peaks of FT-IR are highlighted in the text with the symbol P (for peak) as a super index. Solubility profile of PNSC in aqueous solution (**B**). Process of PNcs size tuning in a range of 90–615 nm (**C**). Inset, TEM image of PNc of 90 nm (a) and 615 nm (b) diameter. Estimation of PNcs and NBc particle size by DLS (D), and surface charge by ELS (**D**, inset), respectively. TEM image of NBc (**E**) and enlarged image (**E**, inset; dotted line). SEM image of lyophilized NBc (**F**) and corresponding size distribution (**F**, inset). The scale bars are 100 nm (black) and 1 μm (white), respectively.

**PAP:** ^1^H NMR (300 MHz, DMSO-D_6_): δ 7.82 (d,4 H), δ 7.56 (m,3 H), δ 6.96 (d,2 H) from phenyl group (Fig. S1A,a, Supporting Information, S.I.); IR (KBr): 3139 cm^−1^ (-OH)^**P10**^, 3060 cm^−1^ (phenyl group)^**P11**^, 1606, 1587, 1505 cm^−1^ (benzene ring)^**P12**^, and 1242 cm^−1^ (-C-O-)^**P13**^ (Fig. 3A**-PAP**); UV/Vis: λ_max_ 356 nm (Fig. S2A, S.I.)^27^.

**PAPE**: ^1^H NMR (300 MHz, DMSO-D_6_): δ 7.91, (t,3 H), δ 7.52 (m,4 H), δ 6.85 (d,2 H) from rings of PAP (Fig. S1B,a, S.I.), δ 4.12 (t,2 H), δ 4.08 ppm (t,2 H), from -CH_2_- (Fig. S1B,b, S.I.); IR (KBr): 3293 cm^−1^ (-OH), 3062 cm^−1^ (phenyl group), 2938, 2872 cm^−1^ (C-H), 1601, 1582, 1497 cm^−1^ (benzene ring) (Fig. 3A**-PAPE**); UV/Vis: λ_max_ 353 nm (Fig. S2B, S.I.)^27^.

**PAPESE**: ^1^H NMR (300 MHz, DMSO-D_6_): δ 7.91 (t,3 H), δ 7.53 (m,4 H), δ 6.86 (d,2 H) from rings of PAPE (Fig. S1C,a, S.I.); δ 4.39 (t,2 H), δ 4.31 (t,2 H), from -CH_2_- (Fig. S1C,b, S.I.), δ 2.50 (2 H) from -CH_2_COOH (Fig. S1C,c, S.I.); IR (KBr): 3062 cm^−1^ (phenyl group), 2931, 2880 cm^−1^ (C-H), 1600, 1584, 1498 cm^−1^ (benzene ring), 1723 cm^−1^ (-CO-), 1417, 1384 cm^−1^ (-COO^−^ ions)^29^ (Fig. 3A**-PAPESE**); UV/Vis: λ_max_ 352 nm (Fig. S2C, S.I.).

**CHI**: ^1^H NMR (300 MHz, D_2_O and TFA): δ 4.50 (H_7_), from –NH_2_ (Fig. S1D,d, S.I.), δ 3.04–3.58 (H_2_, H_3_, H_4_, H_5_), from ring of chitosan (Fig. S1D,e, S.I.), δ 2.85 (H_1_), from -CH_2_NH_2_ (Fig. S1D,f, S.I.), δ 2.39 (3 H), from CH_3_-CO-NH- due to acetylation of chitosan (Fig. S1D,g, S.I.); IR (KBr): 3355 cm^−1^ (-OH)^**P1**^, 2863 cm^−1^ (-CH-)^**P2**^, 1645 cm^−1^ (-CO-NH-)^**P3**^ 1575 cm^−1^ (-NH_2_)^**P4**^, 1150 cm^−1^ (-C-O-C-)^**P5**^, 1026 cm^−1^ (-C-O-)^**P6**^ and 891 (pyranoid ring)^**P7**^ (Fig. 3A**-CHI**)^17,30,31^.

**NSC**: ^1^H NMR (300 MHz, D_2_O and TFA): δ 4.3 (H_7_), from –NH_2_ (Fig. S1E,d, S.I.), δ 3.05–3.59 (H_2_, H_3_, H_4_, H_5_), from ring of CHI (Fig. S1E,e, S.I.), δ 2.86 (H_1_), from –CH_2_NH_2_ (Fig. S1E,f, S.I.), δ 2.36 (3 H), from CH_3_-CO-NH-, (Fig. S1D,g, S.I.), δ 2.42 (2 H) (Fig. S1E,h, S.I.), from -NHCOCH_2_-, δ 1.92 ppm (2 H), from -CH_2_- of succinic anhydride (Fig. S1E,I, S.I.); IR (KBr): 3256 cm^−1^ (-OH), 2972, 2947 cm^−1^ (-CH-), 1657 cm^−1^ (-CO-NH-), 1552 cm^−1^ (-NH-)^P9^, 1401 cm^−1^ (-COO^−^ ions)^P8^, 1153 cm^−1^ (-C-O-C-), 1022 cm^−1^ (-CH-O-) and 921 (pyranoid ring) (Fig. 3A**-NSC**)^17,31^.

**PNSC**: ^1^H NMR (300 MHz, DMSO-D_6_ and TFA): δ 8.23 and (3 H), δ 8.12 (4 H), δ 7.94 (2 H) from rings of PAPESE (Fig. S1F,a, S.I.), δ 3.32–3.64 (H_2_, H_3_, H_4_, H_5_), from ring of CHI (Fig. S1F,e, S.I.); δ 2.88 ppm (H_1_), from -CH_2_NH_2_ (Fig. S1F,f, S.I.), δ 2.41 (2 H) (Fig. S1F,h, S.I.), from -NHCOCH_2_-, and δ 1.89 (2 H) (Fig. S1F,I, S.I.), from -CH_2_- of succinic anhydride; IR (KBr): 3326 cm^−1^ (-NHCO-)^**P18**^, 2936, 2878 cm^−1^ (C-H)^**P14**^, 1630 cm^−1^ (-C-O-)^**P19**^, 1577 cm^−1^ (-NH-)^**P9**^, 1382 cm^−1^ (-COO^−^ ions)^**P8**^, 1600, 1588, 1495 cm^−1^ (benzene ring)^**P12**^, 1147 cm^−1^ (-C-O-C-)^**P5**^, 1026 cm^−1^ (-CO-)^**P6**^ and 918 cm^−1^ (pyranoid ring)^**P7**^ (Fig. 3A**-PNSC**); UV/Vis: λ_max_ 331 nm (Fig. S2D, S.I.).

### Synthesis of loaded-nanobioconjugate

#### PNSC solubility test

Solutions of equal concentrations of PNSC (10 mgml^−1^) were prepared at twelve different pHs (1–12): solid samples of PNSC (200 mg) were added into flasks containing distilled water (20 ml), pH was adjusted previously with aqueous HCl and NaOH and the samples were placed under agitation during 24 h. After that, 12 pieces of filter paper were weighted and the solution filtered through vacuum by slowly adding the mixture into a Buchner Funnel. The filter paper was removed and the volume of the separated liquid measured. Filters were placed on top of Petri dishes and dried at 60 °C overnight. After that, dried filters were weighed to establish their final mass. The final pH was also measured. We used the **equation S1, (**S.I.**)** to obtain the saturation concentration of PNSC in the water at different pHs.

#### Assembly of photosensitive nanocarriers

First, a homogeneous stock of PNSC (2 mgml^−1^) in deionized water was made. The pH level of the water was the one in which higher solubility was obtained in the previous test. A series of solutions with various concentrations (0.6, 0.8 and 1.0 mgml^−1^) was then obtained by diluting the stock solution with water at the same pH. Then, THF (v/v, 1:1, PNSC solution: THF) was added dropwise and slowly. Subsequently, an aqueous solution of water and THF was dripped and the effect of water/THF proportions: 50/50; 80/20 and 90/10%v/v were evaluated. Finally, the PNcs were obtained by slow evaporation of the THF (Fig. S3, S.I.) The PNcs were washed three times by centrifugation at 5000 rpm for 15 min, resuspended in deionized water and stored at 4 °C. The cardiac targeting peptide (CTP) has been selected herein as the cell-targeting biomolecule of the NBc for its demonstrated capacity to disrupt the cell membrane from the cardiac cells. The NBc was obtained by functionalizing the PNcs with the CTP by modifying the methodology published by Sehgal^32^ and Lu (2010) as follow^33^. EDC and NHS were used to activate the carboxyl-terminal groups from the outermost PNcs surface, for conjugation with primary amines from the peptide. Such activation generates a succinimide derivate, that being a very good leaving group is spontaneously replaced by the amino moiety from the CTP. The reaction is performed in 2 steps as detailed as follows. Activation of the carboxylic groups on the surface of PNcs was achieved by incubation of 50 µl of the as-prepared PNcs (2mgml^−1^) in 100 µl of a phosphate buffer saline (PBS) solution prepared by diluting 1.9 mg EDC (20 mM) and 2.1 mg NHS (40 mM) in 500 µl of 25 mM HEPES buffer solution pH 6.5, for 30 min and washing once with 100 µl of HEPES buffer. Functionalization of the FITC-CTP with activated carboxylic groups from the PNcs, by incubation of 100 µl of the activated PNcs in 0.05 M phosphate buffer saline solution (PBS), pH 9.8, containing 250 µgml^−1^ FITC-CTP, for 2 h. Washing the functionalized PNcs with 100 µl of pH 7.4, PBS (1x), containing 0.05% tween 20 and store at 4 °C until use. To perform the experiments, the stored solution is centrifuged at 5000 rpm and resuspended in 100 µl pH 7.4, PBS (1x) (without tween). This functionalization process is depicted in Fig. S4, (S.I.**)**.

#### Concentration of PNcs and FITC-CTP into the NBc

FITC-linked CTP was used to facilitate its characterization by UV- spectrophotometry. The UV spectrum of the PNcs and FITC-CTP were recorded in order to determine the maximum absorbance wavelength. Absorbance-concentration dependence curves were recorded by spectrophotometry using a Multiskan GO UV/VIS, in a wavelength ranging from 200 to 1000 nm. From the spectra, the maximum intensity values obtained at 343 and 492 nm for the PNcs and FITC-CTP, respectively were used to plot two calibration curves that show the corresponding absorbance-concentration dependence. An excess of CTP was used in the reaction to assemble the NBc, named the initial solution. After the reaction, the remained not functionalized CTP was removed by centrifugation at 5000 rpm for 10 minutes. Both, the resultant NBc and the free FITC-CTP in the supernatant were then collected. The pellet was resuspended in a known volume to quantify the concentration (Fig. S5, S.I.) by means their respective absorption-concentration equations (S2 and S3, S.I).

#### Co-encapsulation process of cargo (Nile red and dofetilide) into NBc

The cargo was solubilized in THF before to start the nanoprecipitation process. After that, the protocol is the same as described in the section “assembly of Photosensitive nanocarriers”, using 0.6 mg ml^−1^ of the PNSC. Then, the loaded PNcs were centrifuged and purified to remove cargo excess using DMSO and dialysis. Finally, the functionalization with CTP was made as described in the section “assembly of Photosensitive nanocarriers”. The non-encapsulated cargo was dissolved in DMSO to calculate the loaded concentration, through an absorbance-wavelength plot built for the co-solubilized dye-drug (Fig. S6, S.I.), using their respective absorption-concentration equations (S4 and S5, S.I).

### Photo-triggered and specific delivery of cargo into cells

Cells with less than 17 passages were maintained in DMEM supplemented with 10% (v/v) fetal bovine serum at 37 °C, in a humidified atmosphere containing 5% CO_2_. Biocompatibility assays were performed when cell cultures were at 80% confluence. Initially, the PNcs (or NBc) were added as treatments, at the same time, in 9 plates with cultivated cardiomyocytes. The cell cultures were characterized by fluorescence microscopy at different cultivation times, from 5 to 14 h for PNcs and 5, 6, 7 and 8 h for NBc (each), respectively. These experiments allowed, on one hand, to establish the minimum time that the PNcs need to be internalized in the cardiomyocytes and on the other hand the affinity of the NBc for the cardiac cells. Each image was taken 3 times for each culture, and the entire experiment was done in triplicate, so that (n = 9). Then, 1 ml of 6000 cells (12-well microplates) were incubated and 100 μL of NBc at 0.15 mg ml^−1^, in the determined times. After, the cells are dyed with 100 μL of ethidium bromide (the cell nucleus is dyed in red color) at 0.2 mg ml^−1^ and 100 μL of DIOC6 (the cytoplasm is dyed in green color) at 40 nM.

A UV-light lamp at 365 nm (Analytik Jena; long-wave; 6 watts; 230 VAC/50 Hz) was used to expose over 15-different solutions with the NBc. Each solution was exposed from cero seconds (blank) to 14 seconds with 1-second intervals. Then, the solutions were characterized by UV-spectrophotometry, where the NBc UV spectrum has the maximum absorbance wavelengths at 331 and 433 nm. Then, the photoisomerization process was verified when the band at 331 nm decreased and the one at 443 nm increased. The isomerization extent was determined with the area under the curve, while the blank solution is taken as a reference point, by using the Image J program.

Loaded-NBc (15.15 ± 1.61 μM dye and 14.57 ± 0.87 μM of the drug in 0.15 mg ml^−1^ of NBc) was internalized into 6000 cells for each well (12-well microplates), for 8 h incubation time. After that, they were irradiated with UV-light at 365 nm for 14-s while the cargo released. Then, an excess of KCl solution (0.56%) was added in each well (12-well microplates) to detach the adhered cells and to perform a cellular lysis process. Then, the cells and cargo delivered from them were centrifuged at 12000 rpm, remaining in the pellet. After taking out the cell medium, DMSO was added and the solution centrifuged again. The cargo staying at the supernatant due to its hydrophobic tendency was measured by UV-vis spectrophotometry and the co-encapsulated dofetilide-Nile red concentration curve (in DMSO) was used to quantify the cargo released intracellularly (Fig. S6, and equations S4 and S5, S.I.).

### *In-vitro* cytotoxicity and phototoxicity assays

To assess the short-term cytotoxic effect of NBc and phototoxicity effect on RL14 cardiomyocytes, a MTT assay was conducted as an indicator of the metabolic competence of the cells^34^. Then, RL14 cells (4 × 10^3^ cells/well, 96-well microplates) were incubated in 10 μl of 20 freshly prepared serial-dilutions (1: 2) of NBc (from 2 to 0 mg ml^−1^), for 24 h. In a similar experiment, the cells were incubated and exposed to UV-light from 0 to 15 s. At the end of the incubation time (in both experiments), 10 μl of MTT solution (5 mgml^−1^) was added to each well. The microplates were then incubated in dark, under constant stirring, for 6 h, at 37 °C. 100 μl of cold isopropanol was then added to each well in order to dissolve formazan crystals, followed by gentle stirring, in a gyratory shaker, for 12 to 24 h. After that, the optical density (OD) was measured with the Multiskan-go spectrophotometer, at a wavelength of 570 nm. To verify the repeatability of the results, three independent experiments were performed in triplicate. Also, positive (dimethyl sulfoxide, DMSO 100%) and negative controls (complete culture medium without cells) were also tested.

For PNSC solubility test, size, concentration, charge, morphological and microstructural characterization of nanocarriers, concentration curve of co-solubilized dofetilide and Nile red, in DMSO and fluorescence microscopy characterization, please go to the Supplementary Information section.

## Results and Discussion

### Synthesis and characterization of the photosensitive copolymer

Figure 1 illustrates PNc synthesis, nanocarrier self-assembly and cargo co-encapsulation. PNc synthesis consists of the introduction of the UV-photosensitive 4-phenylazophenol (PAP) molecules into the backbone of a NSC biopolymer to form the amphiphilic copolymer PNSC, which will be the responsible for cargo photo-release when assembled within the PNcs. The synthesis process is followed by self-assembly of nanocapsules via the nanoprecipitation method, through which the PNSC is solubilized in an aqueous solution and dispersed through a drip system in a hydrophobic solvent. Then, while the hydrophobic solvent is being evaporated, the hydrophobic components of the PNSC are aggregated and PNcs formed (Fig. 1 and Fig. S3, S.I.)^35^. The resultant PNcs have a core-shell micellar structure with a hydrophobic core (the PAP) and a hydrophilic surface (carboxylic groups from NSC). They have the ability to transport a hydrophobic cargo inside the core-shell and can be functionalized with biomolecules on their outermost surface^36^, maintaining stability when dispersed in an aqueous medium. When the PNcs are exposed to 365 nm UV light, they suffer a photoisomerization process by increasing the dipole moment of PAP. Therefore, as PNcs size decreases, the polarity changes from a *trans*-PNSC hydrophobic center to a *cis*-PNSC hydrophilic one (Fig. 2)^37–39^. With this conformational change, the PNcs are destabilized and the hydrophobic cargo is released (Fig. 1)^19^. It is important to highlight that the PAP has been linked to poly(acryloyl chloride), to produce a UV-photosensitive amphiphilic copolymer^27^ and some UV-photosensitive micelles^19^. Moreover, UV and NIR-nanocarriers based on chitosan have been synthesized to perform photo-triggered delivery of cargo through the photocleavage mechanism^17,21^. Furthermore, NSC has been extensively synthesized to produce gels and nanoparticles for biomedical applications^40,41^. However, to the best of our knowledge, this is the first conjugation of NSC with PAP (chromophore molecule) to synthesize a new amphiphilic copolymer (PNSC) with the ability to form PNcs for cargo release (Fig. 2).

PNSC synthesis reaction steps were characterized through FT-IR (Fig. 3A). Initially, NSC was synthesized by the reaction between chitosan (CHI) and succinic anhydride (Fig. 2). The characteristic FT-IR peaks of CHI (Fig. 3A**-CHI**) remains in NSC FT-IR spectrum, and the formation of new FT-IR peaks at 1401 cm^−1^ (-COO^−^ ions)^**P8**^ and 1552 cm^−1^ (-NH-)^**P9**^, as well as the vanishing of the peak at 1566 cm^−1^, indicate the proper synthesis of the NSC (Fig. 3A**-NSC**)^31^. In the next step, which links PAP with NSC, PAP was first derivatized to PAPE and later to PAPESE. For this purpose, PAPE was initially obtained by the Williamson ether synthesis, in which 2-chloroethanol is linked to PAP to distance the -OH group from the phenyl ring. Such a distance helps to keep -OH group reactivity, protect -N = N- groups and preserve their photosensitivity properties (Fig. 2)^28^. The characteristic peaks of PAP (Fig. 3A**-PAP**) remain in PAPE FT-IR spectrum. Additionally, the peaks at 2938 and 2872 cm^−1^ (-C-H)^**P14**^ and the –OH^**P15**^ group that shifts towards 3293 cm^−1^ (Fig. 3A**-PAPE**) evidence PAPE formation^27^. Later, PAPESE was obtained from PAPE by an addition reaction in which the succinic anhydride ring opens and its -OH end group links to PAPE through an esterification reaction, whereas its –COOH end group remains free to serve later for linking to NSC (Fig. 2). As a probe of the reaction, it was observed that the PAPESE FT-IR peaks are identical to those of PAPE, but the peak at 3293 cm^−1^ (-OH) vanished and two new peaks at 1723 cm^−1^ (-CO-)^**P16**^ and 1417 cm^−1^ (-COO^−^ ions)^**P17**^ appeared (Fig. 3A**-PAPESE)**. Finally, PNSC was obtained by conjugation of NSC with PAPESE, through a carbodiimide-mediated amidation (Fig. 2). PNSC shows the principal FT-IR peaks of NSC and PAPESE, and the principal peak of amidation is sharper, *i.e*., 3326 cm^−1^ (-NHCO-)^**P18**^, 1630 cm^−1^(-CO-)^**P19**^ (Fig. 3A**-PNSC**). Overall, the FT-IR results demonstrated the proper synthesis of the PNSC molecule.

PNSC synthesis reaction steps were characterized through ^1^H NMR (Fig. S1, S.I.). When comparing the ^1^H NMR spectrum of CHI (Fig. S1D, S.I.) and NSC (Fig. S1E, S.I.) in DMSO-D_6_ and TFA, the new signals at 2.371 and 1.915 ppm are characteristics of amidation between CHI and succinic anhydride, demonstrating the formation of NSC^17^. Comparing the PAP (Fig. S1A, S.I.) and PAPE (Fig. S1B, S.I.) ^1^H NMR spectrums, the new signals in PAPE at 4.120 and 4.08 ppm are characteristics of –CH_2_CH_2_OH, confirming the proper linking of PAP and 2-chloroethanol^27^. For formation of PAPESE (Fig. S1C, S.I.) from PAPE, the ^1^H NMR spectrum presents a new signal, representative of the ester group at 2.503 ppm. Finally, the PNSC ^1^H NMR spectrum (Fig. S1F, S.I.) presents the principal signals of PAPESE and NSC, along with the signal at 2.410 ppm, characteristics of amidation between PAPESE and NSC. The proportion of different side chains ratio of PNSC were estimated from the FT-IR absorbance of CHI, NSC and PNSC polymers (Fig. S7, S.I.) by calculating the percent of free amines (equation S7, S.I.) and using the Table S1 from S.I. and equation S8 from S.I. to determine the percentages of the other polymer segments (Table S2, S.I.), *i.e*. *m* = 10.18%, *k* = 10.06%, *n-m-k* = 63.17%. Thus, PAPESE and succinic anhydride are in the same proportion in the PNSC (10%) (Table S2, S.I.), which led to homogeneous micelles.

### Self-assembly of the photoresponsive nanocarriers

PNcs self-assembly is expected to be more efficient when the polymer is completely solubilized. To reach maximum solubility, we studied the solubility profile of PNSC in an aqueous solution at different pHs. A gravimetric method was then employed to calculate the saturation concentration by means of the equation S1, S.I. The pH of the PNSC solutions was also measured for each saturation state. The transient profile at pH 5 indicated the maximum solubility, which agrees with its buffering capacity pH^42^ (Fig. 3B – blue line). In addition, it was verified that, at pH 5, the highest solubility extent is shown (Fig. 3B - black line). Thus, this pH value was selected for further experiments. Once PNSC was solubilized, PNcs were self-assembled by the nanoprecipitation process (Fig. 1 and Fig. S3, S.I.). PNcs size can be modulated according to the organic solvent: aqueous face ratio (tetrahydrofuran: water at pH 5) and the concentration of PNSC (Fig. 3C). Thus, particle size could be tailored on demand from 615 nm (Fig. 3C,a) to 90 nm (Fig. 3C,b) demonstrating the great versatility of the approach reported herein for a variety of intra- and extra-cellular applications. Size modulation has a direct effect on loading capacity, interaction with different cells, phagocytosis of different cell types, and blood circulation time. A cargo loading capacity of 0.15 mgml^−1^ NBc was estimated to be 15.15 ± 1.61 μM dye and 14.57 ± 0.87 μM drug (Fig. 1) when co-encapsulated. Moreover, the carboxylic groups at the surface of PNcs are amenable to functionalization with CTB for cellular specific photo-triggered delivery, as shown below. Thus, PNcs size and CTB can be selected according to the required application. Herein, PNcs of around 100 nm size are required to pass through the cellular membrane of cardiomyocytes^43^. PNcs were further functionalized with the CTP through an amidation reaction between the carboxyl-terminal groups from the outermost PNcs surface and the primary amines from the CTP (Fig. S4)^32,33^. The CTP has shown to be able to transduce cardiomyocytes rapidly, specifically and efficiently with very little uptake by other cells^44–46^. The average dynamic diameter of the PNcs was 91.00 ± 4.23 nm, which increased by about 10 nm after the functionalization process (Fig. 3D) but is still optimal for cardiomyocytes uptake. Zeta potential changed from −45.4 ± 3.1 mV (PNcs) to −17.1 ± 3.6 mV (NBc) after the functionalization process. The more negative zeta potential of PNcs is caused by the presence of carboxylic groups at the outermost PNcs surface which, mostly ionized, accounts for their stability in aqueous solutions. When coupled to the CTP, primary amines from these molecules make the NBc have a relatively more positive charge (Fig. 3D-inset).

Size, morphology, and functionalization process were studied by TEM and SEM images. Fig. S8, S.I. shows histograms of the particle size distribution from the TEM images, from which the mean particle size was estimated to be 84.06 ± 33.06 and 99.07 ± 43.32 nm for the PNcs and NBc, respectively. Results show not only that the NBc has an apparent slightly higher size with respect to the bare nanoparticles (PNcs and NBc), but also its sizes were slightly smaller than those estimated by DLS, as expected. This apparent discrepancy is related to the fact that whereas DLS shows the hydrodynamic particle diameter, TEM allows for a direct estimation of the geometric particle diameter. In any case, functionalized NBc showed a higher size with respect to the concomitant PNcs counterparts, which indicates that the functionalization process was indeed achieved. The depicted core/shell-like resultant structure is another indication that the PNcs were properly functionalized with CTB. The darker inner part corresponds to the more concentrated core of the polymeric particle, coming from the staining adsorbed by the polymer structure, whereas the clearer thinner outer portion is the peptide. Functionalized PNcs are completely covered by a “halo” that indicates the surface coverage extent is high (Fig. 3E), as compared to the smooth surface of the bare PNcs (Fig. 3Cb). Although their size (as estimated from SEM images) was slightly lower (67.90 ± 13.79 nm), which might be related to the lyophilization process needed for SEM imaging, their spherical morphology was maintained (Fig. 3F). It is important to remark that SEM imaging doesn’t require metal coating because of the presence of resonant π-electrons from the azobenzene groups in the NBc, thus demonstrating the presence of the photosensitive molecule in the NBc structure. Self-fluorescence of PNSC with a maximum absorption peak at 343 nm and fluoresce of FITC-CTP with a maximum at 492 nm, along with their initial concentrations were used to estimate the PNcs and CTP extent in the NBc by means of equations S2 and S3 (S.I.). The resultant NBc has a concentration of 0.15 mgml^−1^ and contains 13.65 ± 2.45 μgml^−1^ of FITC-CTP, which corresponds to 95.63 ± 3.28 µg FITC-CTP mg^−1^ PNcs (Fig. S5, S.I.). This concentration seems reasonable, taking into account that the CTP is a thin layer covering the surface of the PNcs, which is enough for internalization of the NBc in the cardiac cells, as shown below.

### Specificity of the nanobioconjugate for target cells

**Figure 4:**
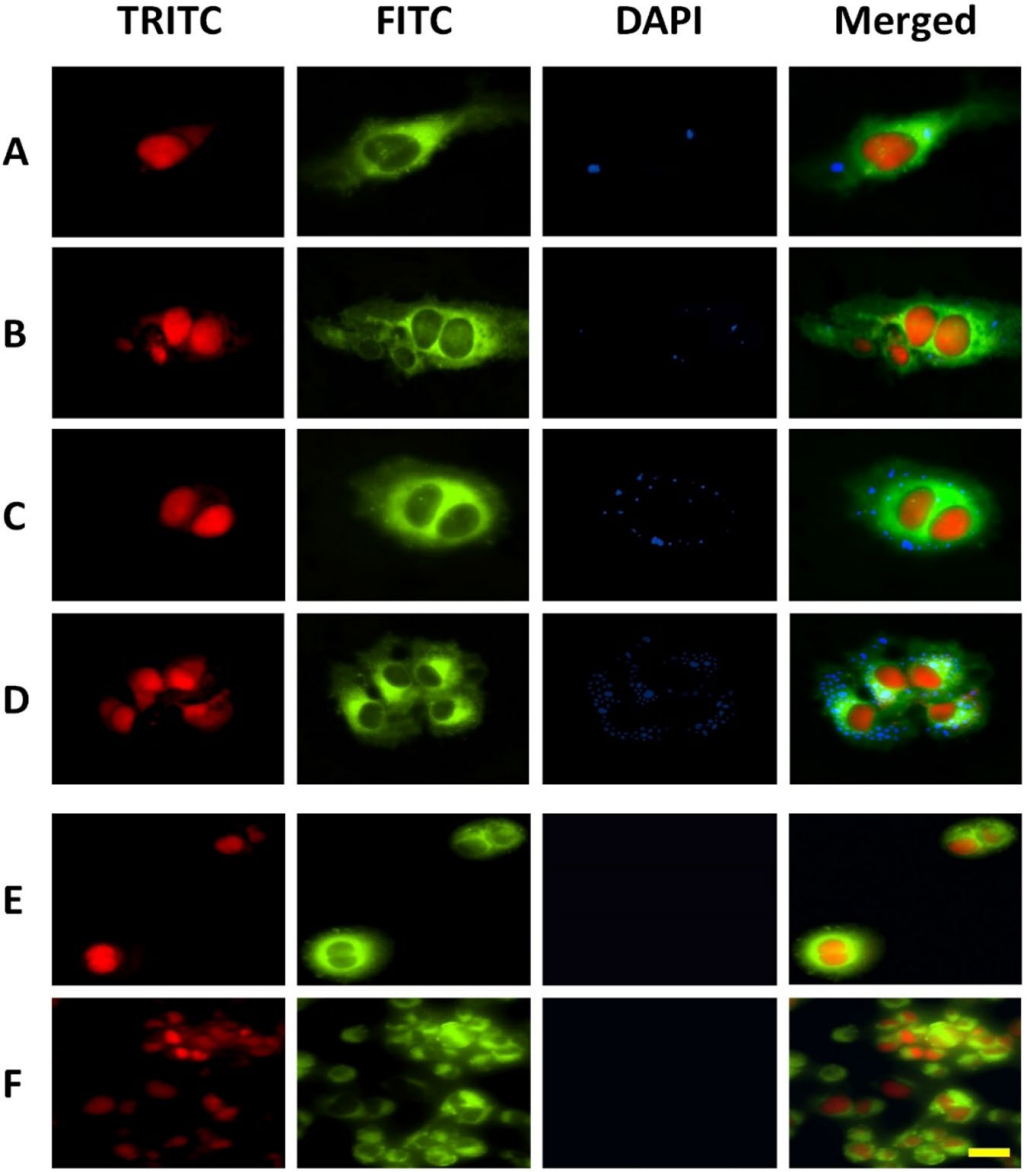
Fluorescence microscopy images of the cell nucleus (stained with ethidium bromide and observed by the TRITC filter), the cytoplasm (stained with FITC), the autofluorescent particles (observed by the DAPI filter) and the three of them (merged). Study the NBc internalization extent into the cardiomyocytes upon time from 6 (**B**), 7 (**C**) and 8 (**D**) h, with respect to the corresponding PNcs incubation for 14 h (**A**), as control of internalization efficiency, and the negligible internalization of the NBc in liver (**E**) and lung (**F**) cells after 8 hours’ incubation, as control of cell specificity. The scale is 25 μm.

To test the specificity of the PNcs functionalized with CTP for the target cells, the resultant NBc was interrogated after incubation in cardiomyocyte cultures for 5–8 h and its fluoresce area compared with 1) those from PNcs (without CTP) after an excess of 14 h incubation as a control of internalization efficiency and 2) those from NBc incubated in liver and lung cell cultures for 8 h used as a control of cellular specificity. Moreover, a design of an experiment carried out by the blocks’ statistical method allowed us to evaluate the incubation times in pairs and to determine the major intracellular internalization time (the higher area occupied by PNcs) (Table S3, S.I.). Results showed that the “hours” (time of incubation variable) have statistically significant differences (F_4,30_=3488.07, P < 0.0001) (Table S3B, S.I.**);** and the “blocks” and the interaction between them “hour-blocks” does not show statistically significant differences (F_2,30_ = 0.00, P = 0.996 and F_8,30_ = 0.88, P = 0.99), respectively (Table S3B, S.I.), which indicates the reproducibility of the experiment. Besides, whereas the NBc does not present internalization in the cardiac cells after a 5-h incubation time, it is slightly internalized after 6-h (Fig. 4B). This is likely a receptor-mediated cellular uptake through the cell bioreceptor-CTP interaction, whose mechanism (endocytic or non-endocytic) is still unclear^46^, but generates an intracellular occupied area of 0.83 ± 0.02% (Fig. S9, S.I.). The increase of their occupied extent in the cardiac cells upon different incubation times (6–8 h) demonstrated the affinity of the NBc for cardiomyocytes, as compared to the internalization extent of PNcs after 14-h incubation used as control (0.81 ± 0.03% of occupied cellular area), (Fig. 4A and Fig. S9, S.I.). Then, 6-h of NBc incubation and 14-h of PNcs incubation are not significantly different (t_30_=0.09, P=0.9291) (Table S3C, S.I.). Yet, the PNcs-CTP (NBc) uptake was 8-h faster than that from PNcs. On the other hand, the internalization extent of the NBc slightly increased up to 1.47 ± 0.05% and up to 15.09 ± 0.09% after 7 and 8 h incubation time with respect to the control (t_30_ = 9.12, P =< 0.0001 and t_30_ = 93.72, P =< 0.0001) (Fig. 4C,D**);** and Fig. S9, S**.I**, respectively, expected as reported by other authors for chitosan-based nanoparticles^47^. The specific affinity was also demonstrated when the NBc was not internalized in liver and lung cells after their incubation for 8-h (Fig. 4E,F). Moreover, the PNcs have little uptake in liver, lung and cardiac cells, at longer incubation time (14-h) (Fig. S10A,C, S.I. and Fig. 4A), and the NBc has little internalization into liver and lung cells at 14-h (Fig. S10B,D, S.I.), consistent with the literature^44,46^. Therefore, 8-h of NBc incubation was the optimal internalization time selected for further experiments (Fig. 4). These results demonstrated the great affinity of NBc for the cardiomyocytes in comparison to the other cells. Based on that we can infer that the CTP is solely responsible for the affinity to cardiac cells and preventing uptake by the lung and liver cells, which would potentially allow a site-specific therapy to decrease the possible side effects of dofetilide on the liver, a concern reported in this kind of therapy^48^.

### Photo-triggered delivery of cargo into target cells and toxicity studies

**Figure 5:**
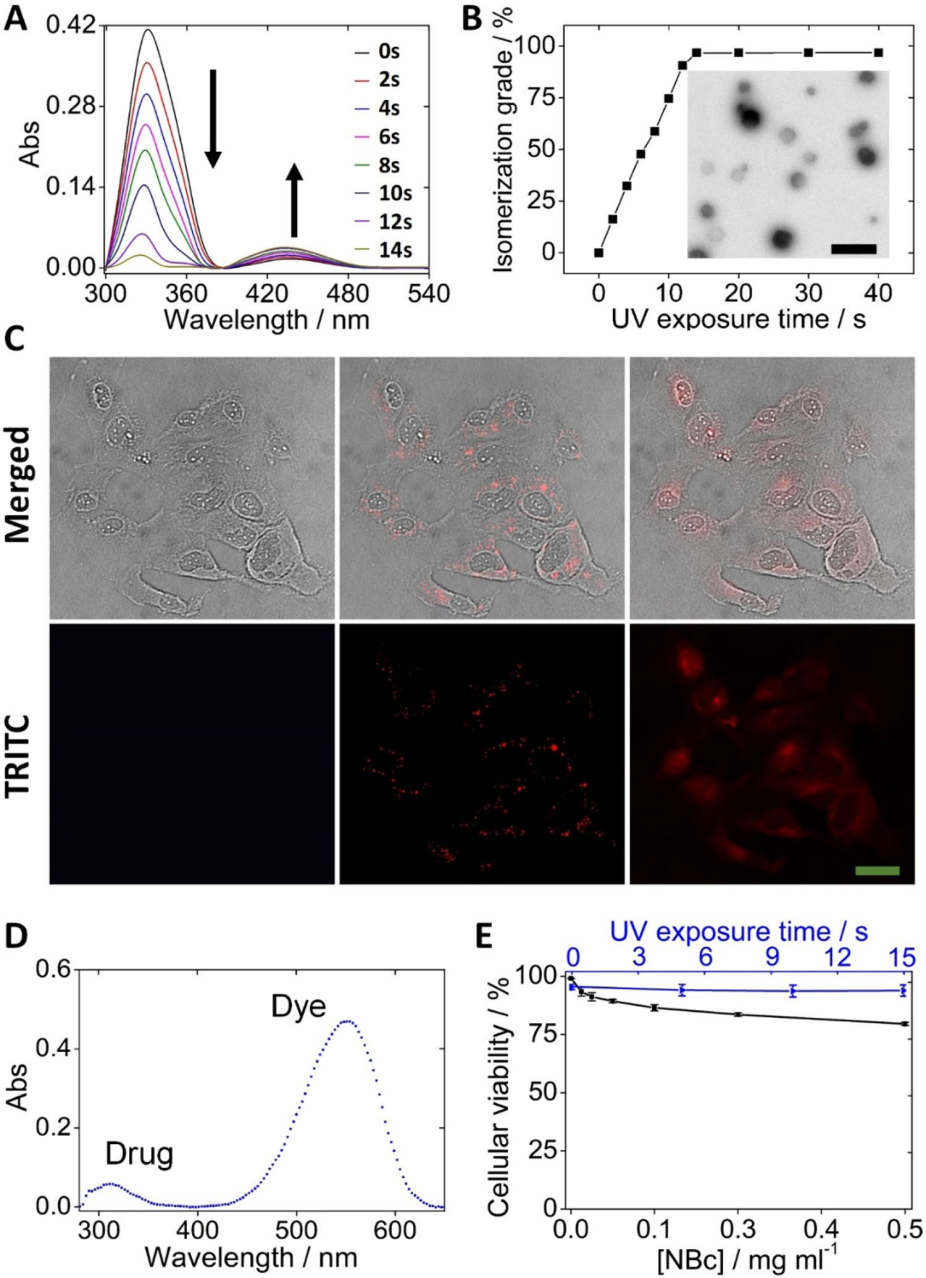
NBc UV-vis spectra variation induced by photoisomerization (**A**), NBc isomerization extent upon UV-exposure time (**B**) and TEM image of NBc after 14 s of UV-exposure (**B**, inset), the black scale is 100 nm. Cargo-specific photorelease process into cardiac cells characterized by fluorescence microscopy. Untreated cells (cardiomyocytes), NBc-encapsulated cargo uptake by the cells at 8 h of incubation and cargo released after 14-s of UV-light irradiation at 365 nm are on the left, central and right columns, respectively. Upper and bottom pictures are the bright field and TRITC filter (Merged), respectively (**C**). The green scale is 100 μm. The concentration profile of co-encapsulated cargo photo-released into cells after cellular extraction (**D**). Cellular viability extent after 24 hours of cell uptake for the phototoxicity (blue line) and cytotoxicity (black line) tests (**E**).

The next set of experiments was conducted to estimate the cargo photo-triggered release into the cardiac cells. For this purpose, we studied the evolution of the UV-vis transition bands from the PAP units (hydrophobic groups from the micelles) upon time, *i.e*. the signal at 331 nm decreasing and signal at 443 nm increasing (Fig. 5A), as an indication of the isomerization process. From these results, cargo photo-delivery was determined to be 97% of isomerization grade, after the NBc was exposed to UV light at 365 nm for 14-s (Fig. 5B). It is important to underline that the isomerization grade is directly related to the cargo-release extent (Fig. S11, S.I). Upon irradiation, the hydrophobic/hydrophilic balance of PAP from the NBc changes, making the molecules rearranges, thus destabilizing the micellar structure and releasing the cargo. Such rearranging, coming from the photoisomerization process (Fig. S12, S.I.), is responsible for the corresponding cargo release. This is clearly evidenced when comparing the TEM images from Fig. 3E with respect to those imagined after the photostimulation process (Fig. 5B**, inset**). The photo-triggered process was characterized through UV-vis spectrophotometry where the dye (Nile red) and the drug (dofetilide) have absorbance maximum peaks at 553 nm and 305 nm, respectively. Moreover, results were contrasted with microscopy experiments, thanks to the dye fluorescence observed with the TRITC-filter (Fig. 5). The cargo-NBc was internalized into the cells after 8-h of incubation (optimum time established), as evidenced by the intracellular red color from the dye (Fig. 5C-center). After cells were exposed to the UV light at 365 nm, both cargos (Nile red and dofetilide) were simultaneously delivered inside cells as inferred from the dye spread in the cytoplasm (Fig. 5C-right). Then, after the cargo was intracellularly released in 6000 cardiac cells, the concentration was estimated to be 13.82 ± 1.47 μM of the dye and 12.14 ± 1.02 μM of the drug (Fig. 5D). This concentration corresponds to 91.24 ± 3.74% and 83.25 ± 2.21% of the dye and drug co-encapsulated into 0.15 mgml^−1^ NBc, respectively.

Cytotoxicity induced by the NBc uptake from the cardiac cell line was assessed after treating the cell cultures with six successive dilutions of NBc from 0 mgml^−1^ (control) to 0.50 mgml^−1^ for 24 h. The cell survival plot showed a dose-dependent response trend (Fig. 5E, black line), where the NBc treatment reduced cell proliferation from 6.66% (0.016 mgml^−1^) to 20.45% (0.50 mgml^−1^), whereas the cell proliferation of untreated cells used as a control was only 0.89% (0 mgml^−1^), respectively. It was observed that cell viability slightly decreased upon increasing NBcs concentration. However, the NBc concentration used for the internalization experiments was only 0.15 mgml^−1^, which showed cellular viability higher than 85%. Furthermore, cell cytotoxicity was tested after 24 h incubation, which is much longer as compared to the NBc internalization time (8 h). Similarly, the phototoxicity of the cell line coming from the UV-light exposure at 365 nm was evaluated with time, with cell survival at 99% for all the studied times (Fig. 5E – blue line). In this context, the NBc and the UV-light would not negatively affect cell viability using the concentration (0.15 mgml^−1^), incubation time (8-h) and exposure time (14-s) selected for our experiments (Fig. 5E). Since biocompatibility of chitosan has been widely reported in the literature^49^ and the CTP does not represent associated toxicity at the concentration used herein, we speculate that the PAPESE molecule -containing an azo compound- may give rise to certain toxicity of the resultant NBc^50^. But the toxicity is minimal due to the low concentration of PAPESE in the PNSC (Table S2, S.I.).

In conclusion, new functional and biocompatible UV-photo-triggered nanocarriers for the localized, cell-specific and fast delivery of cargo have been developed. The nanocarriers were obtained through a methodology that involved the synthesis of a new polymer, self-assembly of nanocapsules and their functionalization with cell-targeting biomolecules. The developed protocol allows for the synthesis of tailor-made nanocarriers with a particle size from 90 to 615 nm, opening a number of possibilities for a variety of applications. Moreover, this is the first PNc functionalized with a cardiac targeting peptide, for the specific photo-triggered antiarrhythmic drug delivery into cardiomyocytes, as a strategy to fight atrial fibrillation, addressing a need from scientists in this field^23,24^. As antiarrhythmic drugs must be delivered only to atrial cardiomyocytes to avoid the proarrhythmic and side effects, this localized therapy could be implemented through a catheter, like in the cardiac ablation-based intervention^51,52^. These smart nanocarriers for fast cargo delivery in specific cells pave the way for a highly specific space-temporal drug delivery triggered by external control, even in a very specific area, depending on how and where the UV-light is applied. The versatility of the PNcs allows them to be used in other types of applications, such as photodynamic therapy and skin lesions, or to treat diseases where the cargo delivery could be controlled by UV-light from sunlight.

## Supplementary information

Supporting information.
