# Supplementary material for: Photosensitive nanocarriers for specific delivery of cargo into cells
Source: Sci Rep. 2020 Feb 7;10:2110. doi: 10.1038/s41598-020-58865-z (PMC7005817; doi:10.1038/s41598-020-58865-z)
Supplement: Supplementary file 1 — Supporting information. [file 41598_2020_58865_MOESM1_ESM.pdf]

SUPPORTING INFORMATION

Photosensitive nanocarriers for specific delivery of cargo into cells

Pedro Mena-Giraldo, Sandra Pérez-Buitrago, Maritza Londoño-Berrio, Isabel C. Ortiz-Trujillo, Lina M. Hoyos-Palacio and Jahir Orozco*

Experimental Section

Protocol for PNSC solubility test: Equation **S1** to obtain the saturation concentration of PNSC in the water at different pHs.

$$C_{sat} = (Initial\ mass\ - Filtered\ mass)/\ Final\ volume \qquad \textbf{(S1)}$$

Where, C_{sat} is the concentration at the saturation point, the *initial mass* is the excess of PNSC polymer mass, the *filtered mass* is the mass of the undissolved PNSC polymer, and the *final volume* is the volume of the filtered solution.

Size and charge characterization of the PNcs and NBc: Particle size distribution and polydispersity index were determined in aqueous solutions, at room temperature, using the Malvern zetasizer Nano-ZS analyzer. Where the hydrodynamic diameters of the PNcs and NBc were calculated according to the Stokes-Einstein equation. Each measurement was done in triplicate, with 100 cycles each. Nanoparticles (PNcs and NBc) surface charge was determined by analyzing samples in aqueous solutions, at room temperature, employing the Malvern Zetasizer Nano-ZS equipment. Each measurement of PNcs and NBc was done in triplicate and the measurements averaged.

Morphological and microstructural characterization: FEI Tecnai G2 20 transmission electron microscope (TEM), Life Science Microscopy Facility and A JEOL JSM 6490 LV scanning electron microscope (SEM) with ultra-high vacuum chamber were used to characterize the morphology of the PNcs and NBc. Nanocarriers (Ncs) were immobilized on top of copper grids, dried at room temperature and stained with phosphate tungsten acid to acquire TEM images. The Ncs were lyophilized and placed on carbon tape to take the SEM images. Moreover, the particle size of Ncs was obtained by analyzing the TEM and SEM histograms acquired through the Image J program.

Concentration of PNcs and FITC-CTP in the NBc: The concentration of PNcs and FITC-CTP could be calculated by using the equations (**S2**) and (**S3**), respectively by subtracting the obtained amount from the initial one.

Figure S5B is a plot of PNcs concentration-absorbance dependence, at 343 nm, ranging from 0.027 to 0.37 absorbance units, described by the linear equation **S2**, with a correlation coefficient (R^2) of 0.9997.

$$\text{C of PNcs}\ \left[\frac{\text{mg}}{\text{ml}}\right] = (\text{Abs} - 0.0044)/0.184 \qquad \textbf{(S2)}$$

Likewise, **Figure S5A** is a plot of FITC-CTP concentration-absorbance dependence, at 492 nm, ranging from 0.036 to 1.0 absorbance units, described by the linear equation **S3**, with a correlation coefficient (R^2) of 0.9998.

$$\text{C of NBc}\ \left[\frac{\mu\text{g}}{\text{ml}}\right] = (\text{Abs} - 0.011)/0.0052 \qquad \textbf{(S3)}$$

Where equation **S2** and **S3** have the form of the following expression $= \frac{y-b}{m}$, being x the concentration, y the absorbance, b the intercept and m the slope of the plots, respectively.

Area extent of PNcs and NBc inside the cardiomyocytes: Fluorescence microscopy images were processed with the Image J program to quantify the cell area extent occupied by NBc and PNcs (treatments) in the cardiomyocytes at different incubation times, described in the section internalization time of PNcs and NBc in cardiomyocytes. Based on that, the cardiac cell area is 100%, and the nanoparticles (NBc and PNcs) area could be determined from.

To have a semiquantitative idea of cell penetration efficiency, the next set of experiments was dedicated to estimating the extent of the cardiomyocytes that were occupied by the PNcs and the NBc. The total cellular area is artificially delimited and estimated using the Image J software (determined as the 100 percent; whereas the nanoparticles (PNcs and NBc), estimated in the same way, correspond to the occupied area of the cardiac cells. The extent was calculated by a conversion factor with values estimated by triplicate, not only in three cells from the same cell culture, n=3, but also from 3 different cell cultures, n=9, to ensure the repeatability and reproducibility intra- and inter-cell cultures (**Figure S9**). Besides, statistical analysis with an ANOVA for a generalized linear model (GLM) was performed to evaluate the differences between treatments, using the two experiments as blocks (random factors within the model) with three replicates per block. A post hoc Tukey-test was developed for multiple comparisons between treatments. This analysis was performed using the statistical package SAS/STAT software version 9.1.3 (SAS Institute Inc., Cary NC 2004) (**Figure S9** and **Table S3**).

Concentration curve of co-solubilized dofetilide and Nile red, in DMSO: Dofetilide concentration-absorbance co-solubilized with Nile red is described by the linear equation S4, by following the peak at 305 nm, ranging from 0.01796 to 0.2188 absorbance units, with a correlation coefficient (R^2) of 0.9976.

$$\text{Dofetilide}\ [\mu\text{M}] = (\text{Abs} - 0.0072)/0.00358 \quad \textbf{(S4)}$$

Likewise, Nile red co-solubilized with Dofetilide is described by the linear equation S5, by following the peak at 553 nm, ranging from 0.0965 to 1.481 absorbance units, with a correlation coefficient (R^2) of 0.9999.

$$\text{Nile red}\ [\mu\text{M}] = (\text{Abs} - 0.0081)/0.03763 \quad \textbf{(S5)}$$

Where equation **S4** and **S5** have the form as in **S3**.

Fluorescence microscopy characterization: Inverted microscope for research with bright field and fluorescence (Ti-U Nikon Eclipse), equipped with a monochromatic camera (Nikon DS-Qi2 16MP USB 3.0), was used for fluorescence microscopy experiments. This microscope was employed to characterize the PNcs and NBc inside of the cardiac cells, liver and lung control cells. Cytoplasm and nucleus of the cardiac cell were stained with DIOC 6, that fluoresces between 495 nm and 515 nm (green) and with ethidium bromide, that fluoresces between 645 nm and 700 nm (red), respectively. PNcs and NBc autofluorescence at 470 nm (blue). The proper fluorescence wavelengths allowed us to visualize the PNcs and NBc internalized in the cardiac cells.

The viability extent (VE): The VE was determined using equation S6.

$$\text{VE} = \frac{\text{OD}_{\text{treated}}}{\text{OD}_{-\text{control}}} * 100\% \quad \textbf{(S6)}$$

Where, OD treated is obtained in the presence of NBc treatments and OD-control is obtained from an untreated sample used as the negative control.

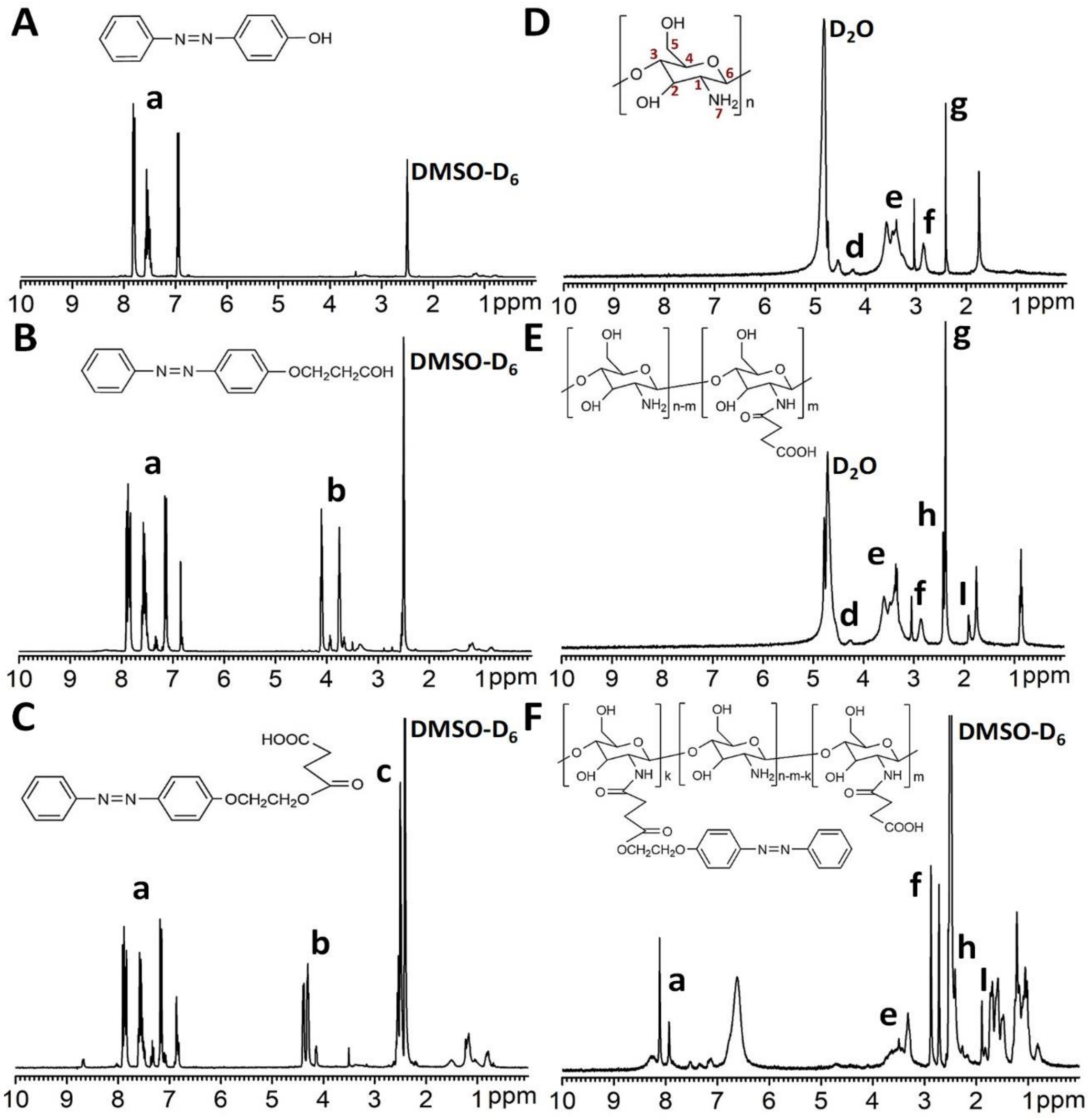

Figure S1. Characterization of the PNSC synthetic route by ^{1}H NMR spectrum of 4-phenylazophenol (PAP) in DMSO-D_6 (A); 2-(4-phenylazophenoxy) ethanol (PAPE) in DMSO-D_6 (B); 2-(4-(phenylazo) phenoxy) ethanoloxy succinyl ester (PAPESE) in DMSO-D_6 (C); chitosan (CHI) in D_2O and TFA (D); N-succinyl-chitosan (NSC) in D_2O and TFA (E); N-succinyl-N-4-(2-(4-(phenylazo)phenoxy)ethanoloxy)-succinyl-chitosan (PNSC) in DMSO-D_6 and TFA (F).

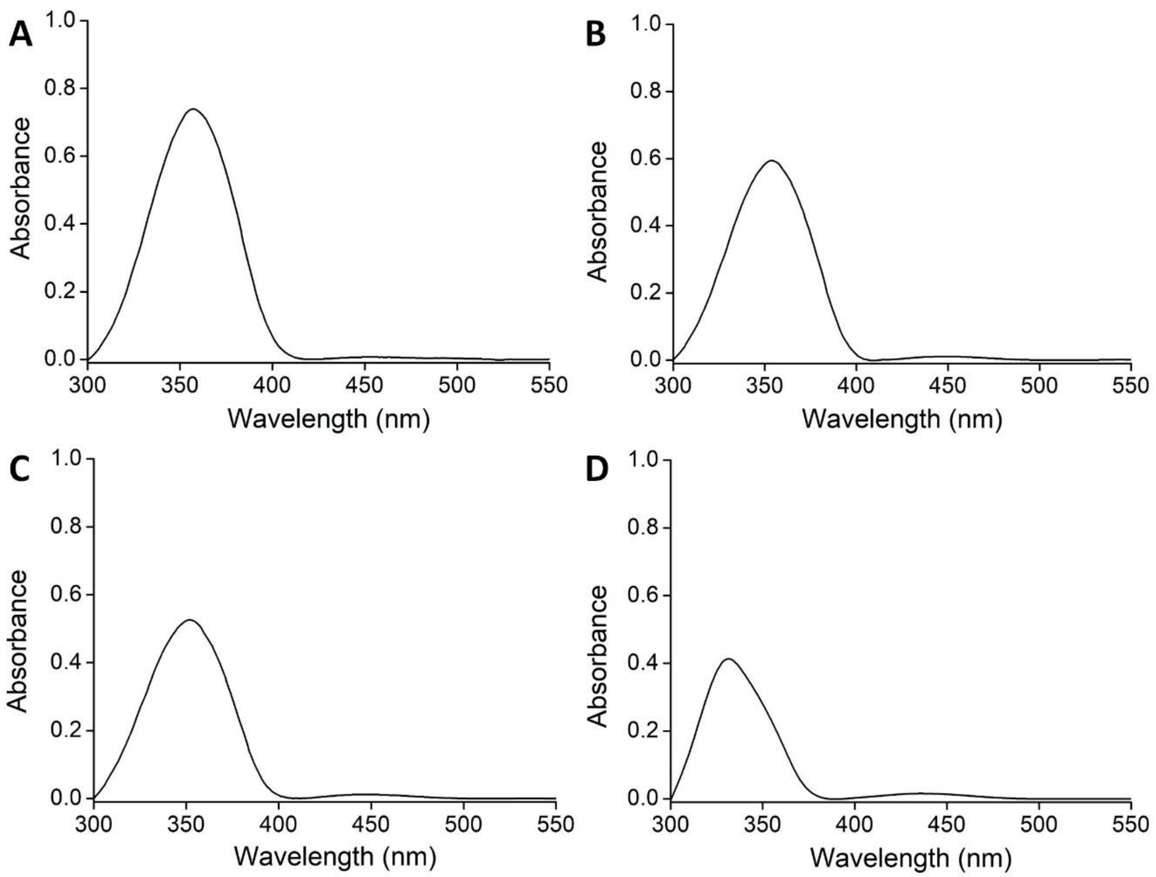

Figure S2. UV/Vis spectrums of PAP (A), PAPE (B), PAPESE (C) and PNSC (D).

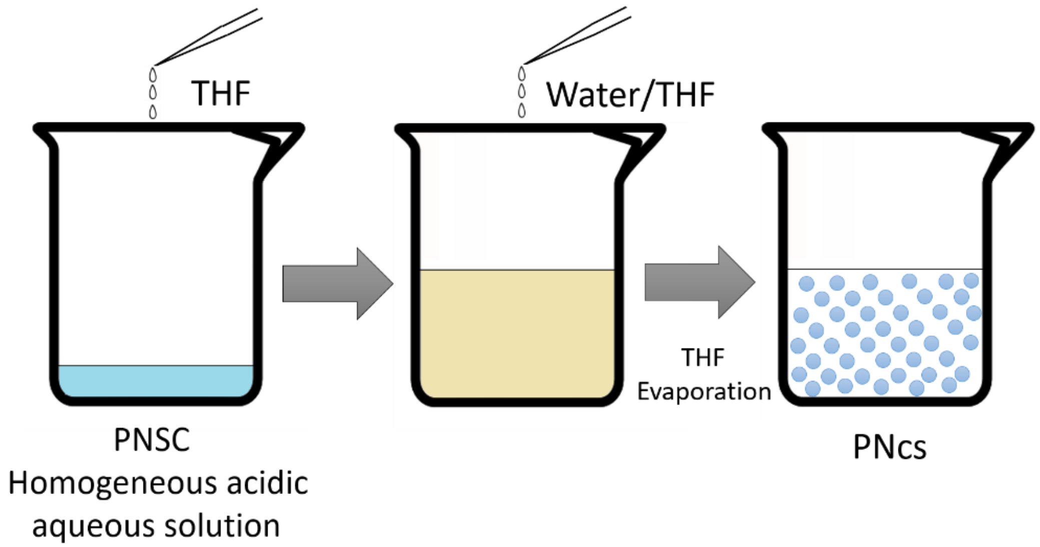

Figure S3. PNSC-nanoparticles formation process.

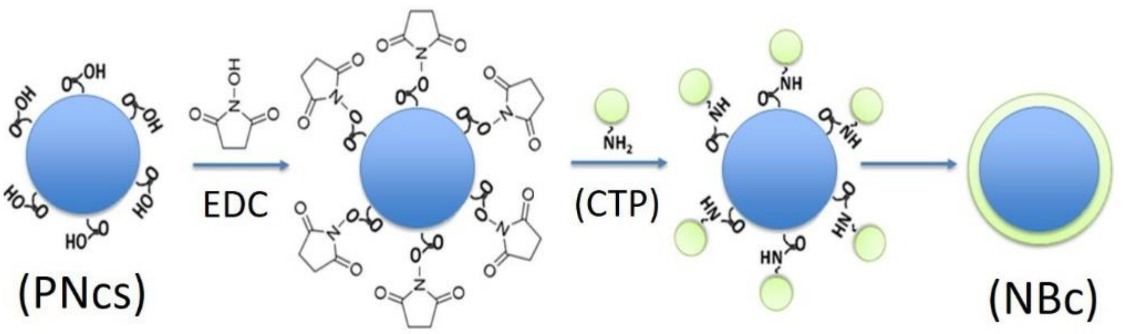

Figure S4. Synthesis of the NBc by covalent coupling the PNcs with the cardiac targeting peptide (CTP) through the EDC/NHS chemistry.

The absorbance peaks at 492 and 343 nm are characteristic of the FITC dye and the self-fluorescence of the NBc-containing polymer, respectively. Therefore, the peak at 492 nm corresponds only to the CTP-FITC. Although the FITC has another peak at 280 nm it does not interfere with the PNcs absorbance peak at 343 nm.[2] Thus, the peak at 343 nm corresponds only to the PNcs. The NBc has been functionalized (PNcs and FITC-CTP) so that they are coexisting in the pellet and therefore the two peaks are observed (**Figure S5**). The supernatant has PNcs (343 nm) and soluble FITC-CTP (492 nm) that was not functionalized.

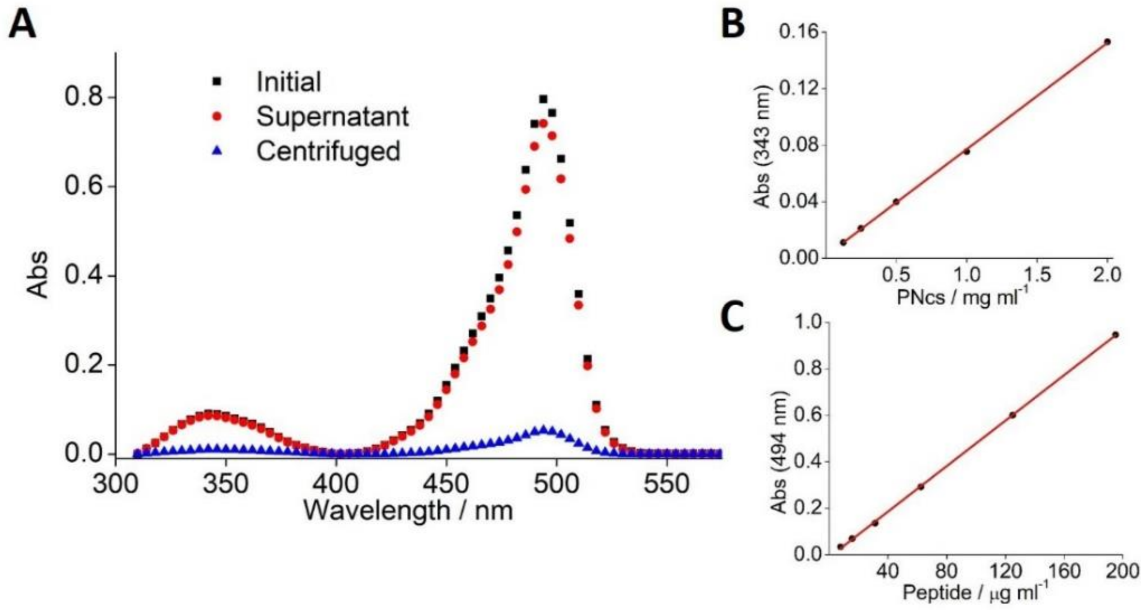

Figure S5. The concentration of FITC-CTP functionalized at the nanobioconjugates surface: Absorbance spectra of the initial solution (black line), the supernatant containing the free FITC-CTP that was not immobilized at the PNcs surface (red line) and the pellet containing the FITC-CTP-functionalized PNcs (blue line) (A). Absorbance and concentration curve of photosensitive nanocarriers (B) and FITC-modified cardiac targeting peptide (C).

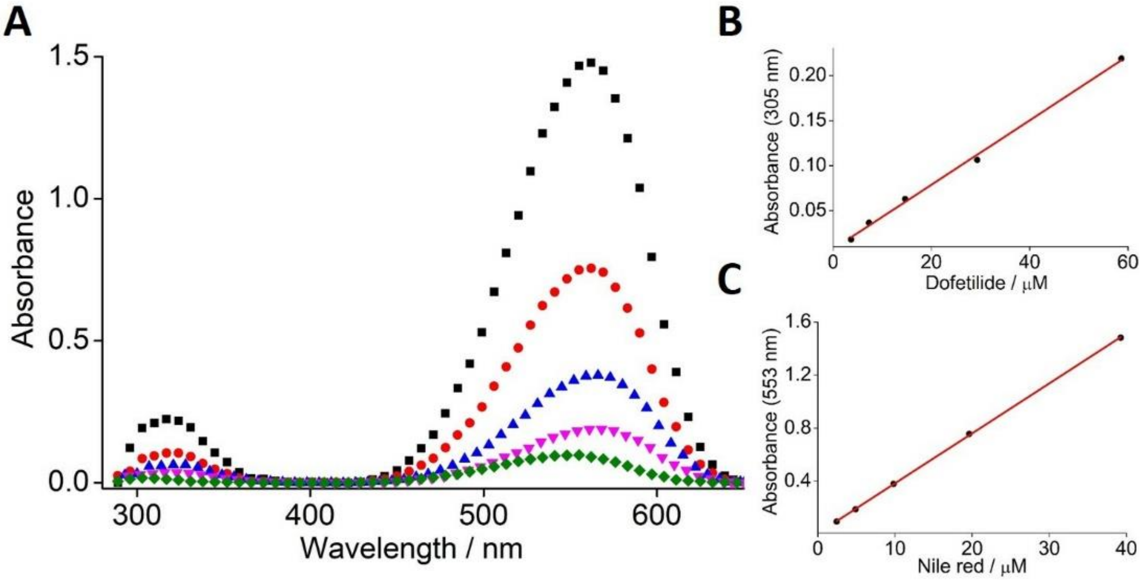

Figure S6. Co-solubilized dofetilide-Nile red absorbance-concentration curve (in DMSO), prepared with 5 dilutions (A), with maximum absorption peaks at 305 nm and 553 nm, respectively. Absorbance-dofetilide concentration curve (B) and absorbance-Nile red concentration curve (C) (in DMSO) when dofetilide and Nile red were co-solubilized.

Quantification of the ratio side chains of PNSC:

To estimate the ratio between different side chains, we use the absorbance spectra of the polymers taken with FT-IR technique and quantify them indirectly with the Baxter method (acetylation from chitosan through FTIR[1]). Then, we can calculate the percent of free amines (%NH_2) from the chitosan polymer (**equation S7**-**table S1**). Such percentage is equal to the deacetylation degree and is directly proportional to the percentage of the polymeric segment with free amino groups (**equation S8**-**table S2**) from PNSC (**Figure A1**).

$$\%NH_2 = 100 - \frac{[A_{1655}]}{[A_{3450}]} * 115 \qquad (\mathbf{S7})$$

Where A_{3450} is the reference band, *i.e.* the absorbance peak corresponding to the evaluation band (amide I) and their limits are between 3900-1850 cm^{-1}. A_{1655} is the evaluation band, *i.e.* the band took as reference (stretching of OH) and their limits are between 1720-1500 cm^{-1} (**Figure S7**).[1]

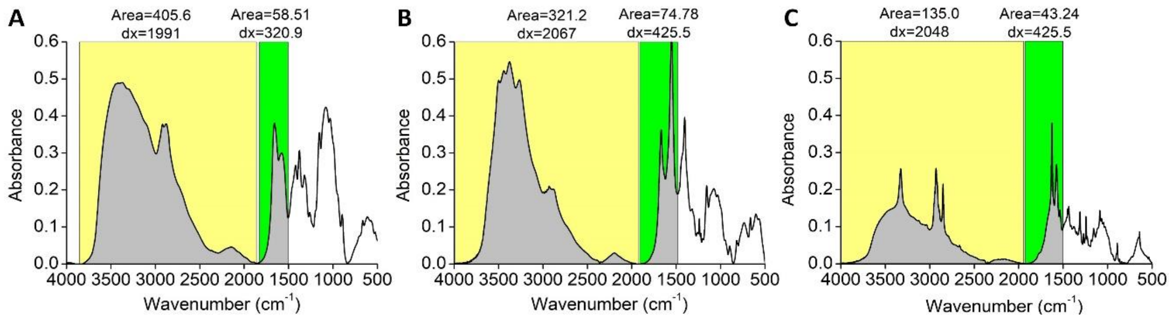

Figure S7. The area down the curve (in color gray) of absorbance spectrum from CH (A), NSC (B) and PNSC (C).

Table **S1** is calculated from equation **S7** and the areas estimated in the figures **S7a-c**, as follows.

Table S1. Free amine percent of the polymers.

Polymer segment	$A_{4000-1800}$	$A_{1800-1500}$	$\%NH_2$
CH (*n*)	405.6	58.5	83.4
NSC (*n-m*)	321.2	74.8	73.2
PNSC (*n-m-k*)	135.0	43.2	63.2

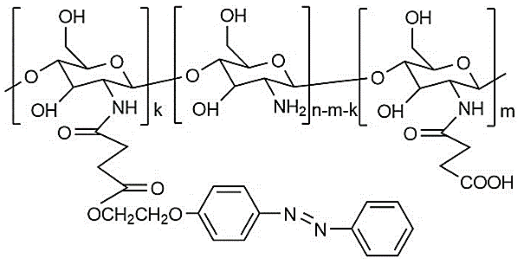

Figure A1. PNSC structure.

The deacetylation degree of chitosan, NSC and PNSC are equal to *n, n-m* and *n-m-k* polymeric segments, respectively. They were estimated by the **equation S8** and listed in Table S2.

$$m = \%NH_{2,CH} - \%NH_{2,NSC};\ k = \%NH_{2,NSC} - \%NH_{2,PNSC} \qquad (\mathbf{S8})$$

Table S2. Polymer segment percent.

Polymer segment	% Polymer segment
n	83.4
m	10.2
k	10.1
n-m-k	63.2

Base on the results, the number of free amines from CH (83.41%) correspond to commercial deacetylation grade (85 %), thus the validity of the method was confirmed. The polymer segments *m* and *k* correspond to the percent of succinic anhydride and PAPESE functionalized in the PNSC, respectively. Thus, PAPESE (inner hydrophobic segment) and succinic anhydride (external hydrophilic segment) are in the same proportion in the PNSC (10%) (**Table S2**), which led to homogeneous micelles. Such proportions resulted to be ideal taking into account that the CTP is functionalized in the succinic anhydride and PAPESE is the molecule that interacts with the drug (impacting the drug loading capacity) and is the responsible of the photo-delivery mechanism. Furthermore, the PAPESE must be minimized to avoid cytotoxicity. It is important to remark that our synthesis process is tunable by linking more PAPESE and/or succinic anhydride groups in the PNSC backbone, thus modulating the drug loading capacity and release mechanism. Herein, it was possible to have the PASESE and CTP in the NBc at limit concentrations.

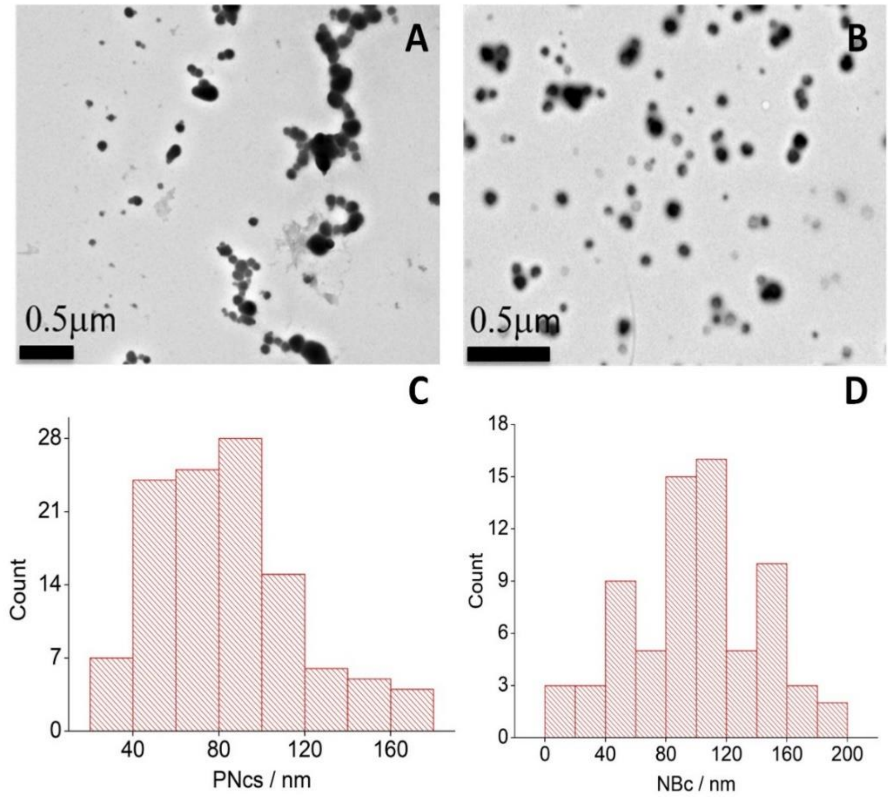

Figure S8. TEM images of PNcs (A) and NBc (B). The histogram shows the size particle distribution from TEM images (C and D).

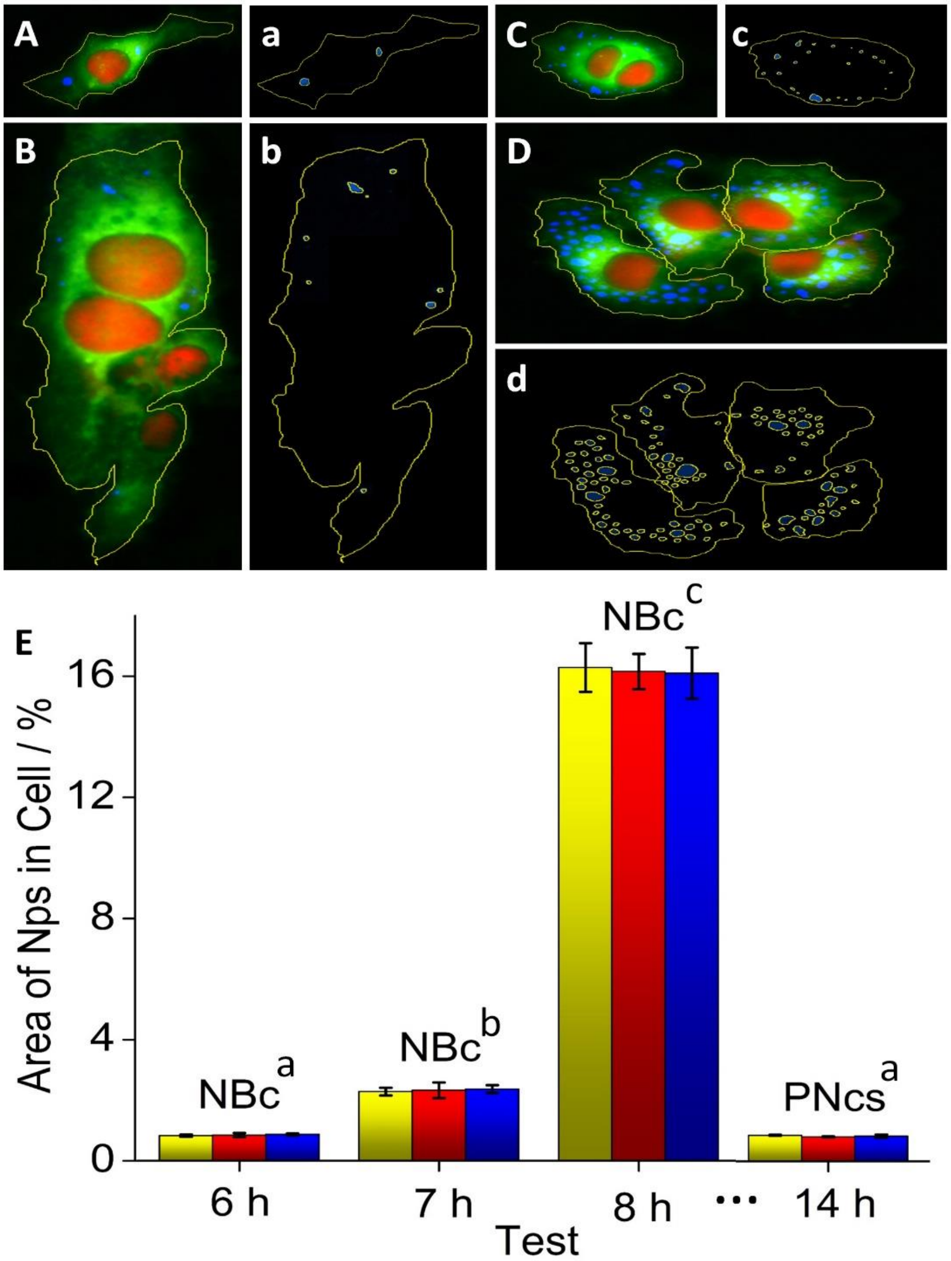

Figure S9. Methodology to estimate the extent of cardiomyocytes that were occupied by PNcs and NBc from the fluorescence microscopy images. Selection of cellular area (A, B, C, and D), PNcs area in Aa, and NBc area in Bb, in Cc, and in Dd, respectively. Nanoparticles extent internalized in the cardyomyocites at different incubation times. The average of triplicate tests at 6, 7 and 8 h (NBc), respect to 14 h (PNcs). The extent was calculated by a conversion factor with values estimated by triplicate from three cells from the same cell culture (n=3), and from 3 different cell cultures, n=9 (E). Different lowercase letters (a, b and c) from E correspond to statistically significant differences with a post hoc Tukey-test with p-values with a level of significance lower than 0.0001 (***). Equal lowercase letters, correspond to no statistically significant differences.

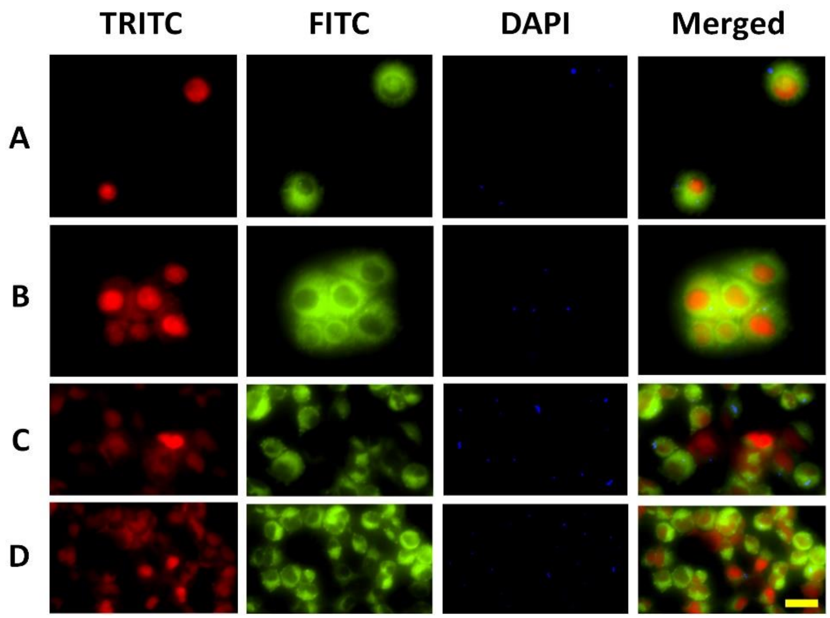

Figure S10. Fluorescence microscopy images of the cell nucleus (stained with ethidium bromide and observed by the TRITC filter), the cytoplasm (stained with FITC), the self-fluorescent particles (observed by the DAPI filter) and the three of them (merged). Negligible internalization of the PNcs (A) and NBc (B) into liver cells and PNcs (C) and NBc (D) into the lung cells, respectively, after incubation for 14 h. The scale is 25 μm.

Table S3. Design of experiments for nanocarriers internalization time in cardiac cells, through a statistical analysis software (SAS).

A. Covariance Parameter Estimates

Covariance Parameter	Estimate	Standard Error
Hours-Block*Rep	0.01217	0.0301
Residual	0.1044	.

B. Type III tests of fixed effects

Effect	Numerator DF	Denominator DF	F	P > F
Hours	4	30	3488.07	<0.0001
Block	2	30	0.00	0.996
Hours-Block	8	30	0.08	0.9996

C. Differences of hour least squares means adjustment for multiple comparisons: Tukey test

Hours	Hours	Estimate	Standard Error	DF	t	P > ItI	Adjusted P
5	6	-0.8286	0.1609	30	-5.15	<0.0001	0.0001
5	7	-2.2813	0.1609	30	-14.17	<0.0001	<0.0001
5	8	-15.8977	0.1609	30	-98.78	<0.0001	<0.0001
5	14	-0.8142	0.1609	30	-5.06	<0.0001	0.0002
6	7	-1.4527	0.1609	30	-9.03	<0.0001	<0.0001
6	8	-15.0691	0.1609	30	-93.63	<0.0001	<0.0001
6	14	0.01444	0.1609	30	0.09	0.9291	1.0000
7	8	-13.6164	0.1609	30	-84.6	<0.0001	<0.0001
7	14	1.4671	0.1609	30	9.12	<0.0001	<0.0001
8	14	15.0835	0.1609	30	93.72	<0.0001	<0.0001

*F: F-statistics parameter, DF: Degrees of freedom, P: Probability, t: t-value parameter

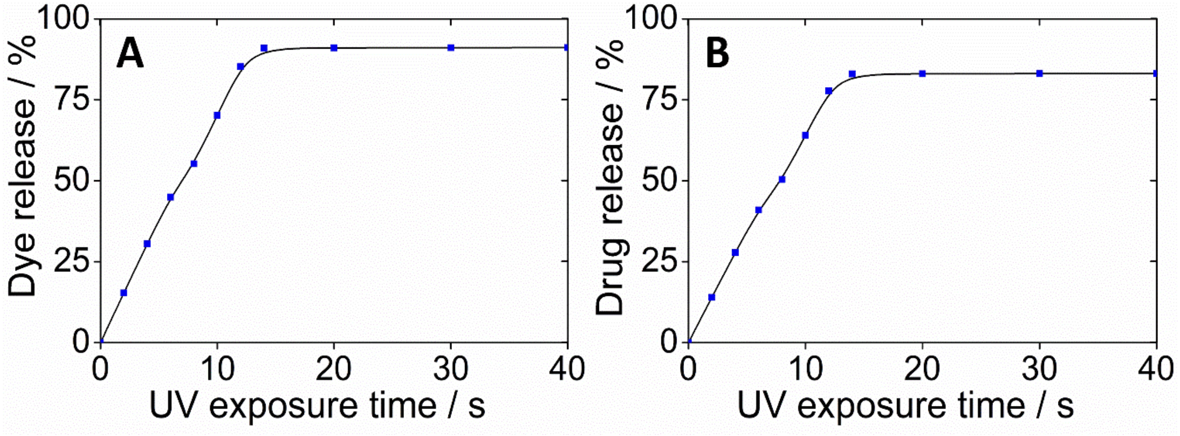

Figure S11. Co-encapsulated cargo release extent upon UV-exposure time, Nile red dye (A) and dofetilide drug (B), respectively.

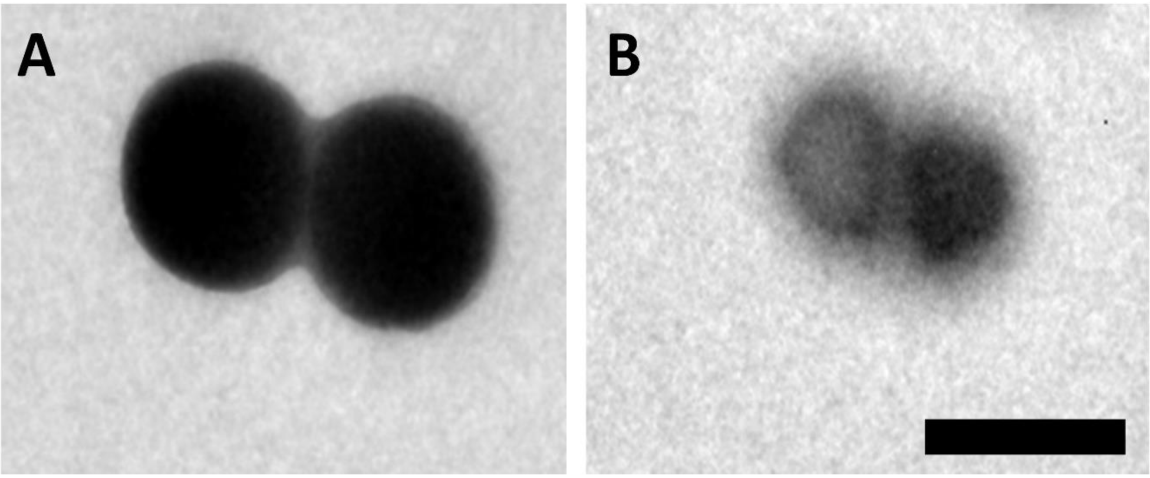

Figure S12. Nanobioconjugated photoresponse process. TEM images of the NBc (A) and the NBc after 14s of UV-light exposure at 365 nm (B), the black scale is 100 nm.

References

1. Baxter, A., Dillon, M., Taylor, K. D. A. & Roberts, G. A. F. Improved method for ir determination of the degree of N-acetylation of chitosan. *Int. J. Biol. Macromol.* **14**, 166–169 (1992).

2. Barbero, N., Barolo, C. & Viscardi, G. Bovine serum albumin bioconjugation with FITC. (2016).
